# Maternal and fetal outcomes of uterine rupture and factors associated with maternal death secondary to uterine rupture

**DOI:** 10.1186/s12884-017-1302-z

**Published:** 2017-04-12

**Authors:** Geremew Astatikie, Miteku Andualem Limenih, Mihiretu Kebede

**Affiliations:** 1 Department of Obstetrics and Gynecology, College of Medicine and Health Sciences, University of Gondar, Gondar, Ethiopia; 2 Department of Midwifery, College of Medicine and Health Sciences, University of Gondar, Gondar, Ethiopia; 3 Institute of Public Health, Department of Health Informatics, College of Medicine and Health Sciences, University of Gondar, Gondar, Ethiopia; 4 Leibniz Institute for Prevention Research and Epidemiology - BIPS, Bremen, Germany

**Keywords:** Ethiopia, Maternal death, Uterine rupture, Ruptured uterus

## Abstract

**Background:**

Maternal mortality and morbidity are the priority agenda for sub-Saharan Africa including Ethiopia. Uterine rupture is the leading cause of maternal and fetal death in developing countries. Limited evidence is available on the magnitude of uterine rupture; maternal and fetal outcomes of uterine rupture and factors associated with maternal death secondary to uterine rupture in Ethiopia. This study aimed to assess the magnitude of uterine rupture; maternal and fetal outcome of uterine rupture and factors associated with maternal death secondary to uterine rupture in Debremarkos Referral Hospital, Northwest Ethiopia.

**Methods:**

An institutional-based cross-sectional study was conducted in December 2015 in Debremarkos referral hospital, Northwest Ethiopia. A total of 242 records of mothers with uterine rupture at Debremarkos referral Hospital during the year 2011–2014 were included in the study. Secondary data was collected from the records of mothers admitted for the management of uterine rupture. Descriptive statistics were performed to characterize the study population. Bivariate and multivariable logistic regression model was fitted to identify factors associated with maternal death secondary to uterine rupture. Odds ratio with 95% confidence interval was computed to determine the level of significance.

**Results:**

A total of 10,379 deliveries were attended A total of 242 uterine rupture cases were included in this study. The magnitude of uterine rupture was 2.44% (1 in 41 deliveries). Sixteen (6.6%) mothers died from uterine rupture. Fourteen (5.8%) had experienced Vesico Vaginal Fistula. The majority of the mothers, 72% (176), admitted for uterine rupture stayed in hospital for 6–10 days. Fetal outcome was grave, 98.3% (238) were stillborn. Place of labor [Adjusted odds ratio (AOR): 6.92, 95% confidence interval (CI): (1.16, 33.74)], occurrence of hypo volume shock [AOR: 3.48, 95% CI: (1.01, 11.96)] and postoperative severe anemia [AOR: 0.092, 95% CI: (0.01, 0.956)] were significantly associated with maternal death secondary to uterine rupture.

**Conclusion:**

The magnitude of uterine rupture was high in the study area. Initiation of labor at health institutions, early treatment of hypo-volumia and prevention of postoperative anemia is recommended to decrease maternal death secondary to uterine rupture.

## Background

Uterine rupture is the tearing of the uterine wall and the loss of its integrity through breaching during pregnancy, delivery or immediately after delivery. It is a catastrophic event in obstetrics, often resulting in both maternal and fetal adverse consequences. Beyond this, it may expose the women have harmful sequel such as permanently infertility secondary to hysterectomy [1].

Maternal mortality remains unacceptably high across many of the developing world especially in Sub-Saharan Africa (SSA) and South Asia. More than 87% of maternal deaths from the global maternal mortality ratio of 210 deaths per 100, 000 live births in 2013 is accounted by Sub-Saharan Africa and South Asia [2]. Ethiopia is the fifth country where the highest maternal mortality occurred next to Democratic Republic of Congo [3]. In this country, uterine rupture and obstructed labor together account for 29% of the total maternal mortality. This makes uterine rupture and obstructed labor to be the second major causes of maternal mortality next to abortion-related complications [4].

Even though uterine rupture is a rare event in developed countries, it is still one of the major public health problem in developing countries that endanger the life of many mothers [5]. WHO systematic review of maternal mortality and morbidity secondary to uterine rupture showed that the prevalence of uterine rupture tends to be lower in developed countries than developing countries with a prevalence rate of 0.006%. Uterine rupture in developed countries mostly occurs secondary to prior cesarean section [1].

Globally, the incidence of uterine rupture is 0.07% which is much lower than what is in Africa - 1.3% [6]. The main reasons for the occurrence of uterine rupture in developed countries are the use of uterotonics and trial of labor on a scarred uterus [7–9].

However, the major causes of uterine rupture in developing countries are obstetric and non-obstetric multihued of factors such as; multi-gravidity, teen-age pregnancy, old primi, poor socio-economic status, previous cesarean section scar, unsupervised labor and unwise use of uterotonic agents [10]. A study from Uganda reported that multi-gravidity, old age pregnancy, and rural residency as significant predictors of uterine rupture [11]. In addition, studies from Nigeria and Uganda showed that the main reasons for the occurrence of uterine rupture were unwise use of oxytocin drug, obstructed labor, grand multi-parity and abnormal fetal presentation [11–13].

A study conducted in Adigrat, Ethiopia showed that the causes of uterine rupture were cephalo - pelvic disproportion, mal-presentation, trial of instrumental delivery, unwise use of Pitocin for induction of labor and trial of labor with previous cesarean section scar. Uterine rupture maternal and fetal case fatality rate was 11.1% and 98.1% respectively [14].

The determinant factors for maternal outcome of uterine rupture differ across geographical boundaries due to the difference in socio-demographic status, and the availability and accessibility of skilled birth attendant and health system effectiveness. Assessing maternal outcome of uterine rupture and factors associated with maternal death in the study area is important to design policies and strategies for the prevention and the clinical management of uterine rupture. Therefore, This study aimed at assessing the magnitude of uterine rupture; maternal and fetal outcome of uterine rupture and factors associated with maternal death secondary to uterine rupture in Debremarkos Referral Hospital, Northwest Ethiopia.

## Methods

### Study setting

Institutional based cross-sectional study was conducted in December 2015 at Debremarkos referral hospital. Debremarkos town is located 295 kilometers from Addis Ababa, the capital of Ethiopia. This hospital is one of the referral hospitals in Amhara Regional State and it potentially serves for more than five million people of the East Gojjam Zone and 4 districts of the West Gojjam Zone. During the study period, one Obstetrician, three Emergency surgical officers, one General practitioner, six BSC and ten Diploma Midwives run the obstetrics and gynecology ward of the hospital. On average, the hospital conducts nearly three thousand deliveries annually.

### Study population

For this study, all records of mothers with uterine rupture who delivered and managed at Debremarkos referral hospital during the year 2011–2014 were retrieved and included in the study.

### Variable of the study

The outcome variables for this study were maternal outcomes, fetal outcomes and maternal death secondary to uterine rupture. Length of hospital stay, admission related outcomes i.e whether the mother admitted due to uterine rupture was improved and discharged from the hospital, maternal death and immediate causes of maternal death were investigated. Fetal outcomes considered in this study were stillbirth, live birth and whether the neonate was improved and discharged from the hospital. Due to numerous missing values, additional fetal outcomes such as Apgar scores and birth weight were not included in our analysis.

We retrieved the charts of uterine rupture cases and collect independent variables such as socio-demographic characteristics (age, parity and place of residence), pregnancy and labor related variables (history of antenatal care, duration of labor, maternal vital signs, previous uterine scar, obstructed labor, and presence of hypovolemic shock during labor.) and treatment-related variables (blood transfusion, abdominal hysterectomy, and uterine repair). Maternal death secondary to uterine rupture was defined as death of the mother from uterine rupture, its complication or management. We also collected data about the presence of complete and incomplete uterine rupture, uterine scar, obstructed labor, induction of labor, and augmentation of labor.

### Data collection procedure

First, the records of mothers with a case of uterine rupture managed at Debremarkos referral hospital from September 2011 to August 2014 were identified using their medical recording number from delivery and operation recording books. Six diploma midwives were selected to collect the data and two BSC midwives were recruited for supervising the data collection process. After 254 charts were retrieved, data was collected using a structured and pretested questionnaire. This questionnaire was used only to acquire data from the medical records. Data were then entered using EPI-INFO version 3.5.3 and exported to SPSS version 20 statistical software for further analysis. Descriptive statistics were carried out to characterize the study population using different variables. Both bivariate and multiple logistic regressions were used to identify factors associated with maternal death secondary to uterine rupture. Variables having a p value of ≤ 0.2 in the bivariate analyses were fitted into a multiple logistic regression model to control the effects of confounding. A p value of less than 0.05 was considered to declare the level of statistical significance in the multivariable logistic regression.

## Results

A total of 10,379 deliveries were managed at Debremarkos referral hospital during January 1^st^, 2011 to December 30^th^, 2014. From this, 254 cases were admitted for uterine rupture.

### Socio-demographic characteristics

Out of the 254 cases of uterine rupture, 242 (95%) charts of women were included in the analysis for this study. The remaining 12 cases of uterine rupture were discarded because of incomplete, damaged and unreadable texts written from the charts.

More than half (56.6%) of the respondents were in the age group of 20–29. Two hundred seven (85.5%) of mothers with uterine rupture were from rural areas. Hundred fifty three (63.2%) of the mothers with uterine rupture had no history of ANC follow up. More than half of the respondents, 138 (57%) mothers started labor at home (Table 1).

**Table 1:** Socio-demographic characteristics of mothers managed for uterine rupture at Debremarkos referral hospital, from 2011–2014. (*n* = 242)

Variable	Number	Percent
Age		
15–19	3	1.2
20–29	137	56.6
30–39	85	35.1
≥40 Marital status	17	7
Married	238	98.35
Divorced	3	1.24
Widowed	1	0.41
Religion		
Orthodox	230	95.04
Muslim	10	4.13
Protestant	2	0.83
Educational status of mothers		
Cannot read and write	170	70.25
Can read and write	50	20.66
Elementary education	20	8.26
Secondary education and above	2	0.83
Ethnicity		
Amhara	236	97.52
Oromo	2	0.83
Tigre	4	1.65
Place of residence		
Urban	35	14.5
Rural	207	85.5

### Prevalence of uterine rupture

**Fig. 1:**
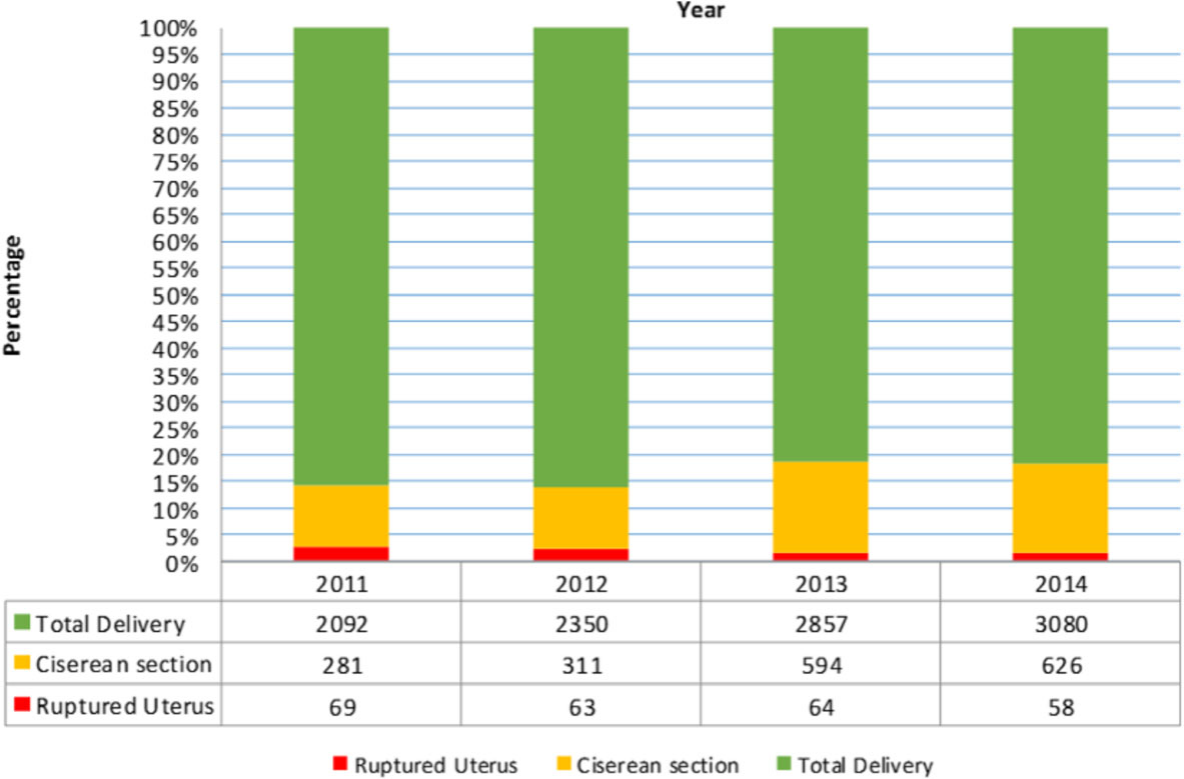
Annual numbers of deliveries, cesarean sections, uterine rupture percentages and uterine rupture: total delivery ratio at Debremarkos referral hospital from 2011-2014 (*n* = 242)

From a total of 10,379 deliveries during the study period, 1812 (17.4%) of them were assisted by cesarean sections and 254 cases were uterine rupture with an overall prevalence rate of 2.44% or 1 uterine rupture in 41 deliveries. The total number of deliveries and cesarean section rate have increased considerably over the four years period. However, the proportion of uterine rupture remained consistent (Fig. 1).

### Obstetric risk factors of mothers who faced uterine rupture

Among the total of 242 cases of uterine rupture, 126 (52.1%) of mothers labored for longer than 24 h, 101 (41.7%) of the mothers labored for 12–24 h and 15(6.2%) mothers labored for 8–12 h. Eighty-nine (36.8%) mothers had no antenatal care follow up and 138 (57%) mothers started their labor at home. The majority, 216 (89.3%), of the mothers presented with obstructed labor, 9 (3.7%) mothers had history of induction-augmentation, 9 (3.7%) mothers had previous history of uterine scar and 8 (3.3%) mothers had trial of instrumental delivery.

### Preoperative clinical presentations of cases with uterine rupture

Two Hundred nine (86.4%) of mothers were diagnosed with uterine rupture preoperatively, 33 (13.6%) of cases were diagnosed intraoperatively. One hundred fourty-two (58.7%) of mothers had abdominal pain and or tenderness, 148 (61.2%) had cessation of uterine contraction, and 177 (73.1%) didn’t have fetal heart sounds, 102 (42.1%) mothers had multiple fetal palpable part, 83 (34.3%) mothers presented with hypovolemic shock, and 37 (15.3%) mothers came with vaginal bleeding. Two hundred eighteen (90.1%) of mothers had preoperative HCT of ≥34, 17(7%) mothers had preoperative HCT value of 22–33% and 7(2.9%) mothers had preoperative HCT value of < 22%.

### Intraoperative findings and surgical procedures of uterine rupture

During laparotomy, the majority of uterine rupture, 208 (86%) have a complete uterine rupture and 34(14%) had incomplete rupture. One hundred seventy-three (71.5%) of ruptures were anterior lower uterine segment, 43 (17.8%) cases were ruptured at the lateral segment and 26 (10.7%) cases had posterior segment rupture. Total abdominal hysterectomy (TAH) was done for 138 (57%) of the cases, for 41 (16.9%) cases subtotal hysterectomy was performed, for 54(23.3%) cases uterine repair with bilateral tuba ligation (BTL) was done, for 9 (3.7%) cases uterine repair without BTL, for 56(23%) cases unilateral salphingo- oophorectomy was done and 24(9.9%) cases had uterine rupture associated bladder rupture and repair was done.

### Postoperative condition of mothers managed for uterine rupture

One hundred twenty-one (50%) of the cases had post-operative hematocrit value (HCT) of ≥34%, 68 (28.1%) cases have HCT value of 22–33% and 53 (21.9%) cases have post-operative HCT value of < 22%. Among those who have uterine rupture one hundred eighty-four (76%) of mothers had received blood transfusion. Among those who received blood transfusion 146 (79.3%) cases were treated with one unit of blood, 31 (16.8%) cases received two units of blood, 5(2.7%) cases received three units of blood, one (0.5%) patient was treated with four units of blood and one (0.5%) patients got five units of blood.

### Postoperative complication of mothers who had uterine rupture

**Fig. 2:**
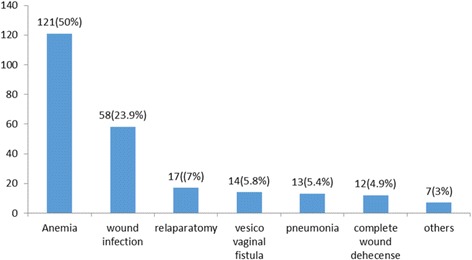
Type and percentage of post-operative complication of uterine rupture at Debremarkos Referral Hospital, Northwest Ethiopia, from 2011–2014

One hundred forty-nine (61.5%) of mothers managed for uterine rupture had developed postoperative complication. Of this, most of them (111 (48.5%)) had developed anemia and 17 (7%) cases had developed surgical site wound infection (Fig. 2).

### Maternal outcomes of uterine rupture

**Fig. 3:**
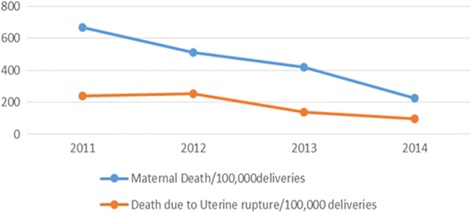
Trend of Maternal Death Vs Death due to uterine rupture at Debre Markos Referral Hospital, 2011–2014

Of the total of 242 mothers admitted for uterine rupture, 226 (93.4%) were discharged and improved. However, 16 (6.6%) of the mothers with uterine rupture died from different immediate causes of death: 6 mothers died from hypovolemic shock, 4 from severe anemia, 3 from septic shock, 1 from acute renal failure and 1 mother from pulmonary edema. One hundred seventy-six (72%) of the mothers have stayed in the hospital for 6–10 days and 44 (18.1%) of them stayed between 11 and 20 days in the hospital. Tragically, the perinatal outcomes were with grave consequences: 238 (98.3%) were stillbirths and only 4(1.7%) were live births. From the four live births, 2 were delivered through instrumental delivery, one from incomplete rupture, and one neonate was delivered from mother having previous cesarean section uterine scar dehiscence (CSD). However, for only one neonate, the discharge information was complete. It shows one neonate was “improved and discharged from the hospital”. As demonstrated in Fig. 3, maternal mortality has significantly declined from 2011 (669 per 100,000 live births) to 2014 (227 per 100,000 live births) (Chi-square for trend = 6.4, p-value = 0.01). More than 35% (14 out of 45) of the maternal deaths registered in Debre Markos Hospital were due to uterine rupture. Moreover, there is no significant decline of maternal death attributed by uterine rupture during the period of 2011–2014.

### Factors associated with maternal death secondary to uterine rupture

Those mothers who had labored at home (AOR: 6.9, 95% CI: 1.16, 13.74), mothers with ruptured uterus who developed hypovolemic shock (AOR: 3.48, 95% CI: 1.01, 11.96) and mothers with uterine rupture who have developed postoperative severe anemia (AOR: 0.092, 95% CI: 0.01, 0.956) were more likely to die of uterine rupture in the multivariate logistic regression analysis.

Mothers with ruptured uterus who labored at home were more likely to die than who labored at a health facility (AOR: 6.9, 95% CI: 1.16, 13.74).

Those mothers with ruptured uterus who developed hypovolemic shock were more likely to die than who did not developed hypovolemic shock after uterine rupture (AOR: 3.48, 95% CI: 1.01, 11.96).

Mothers with uterine rupture who did not developed postoperative anemia (postoperative HCT value of ≥ 22%) have 91% less risk of death than those mothers with uterine ruptured who developed postoperative anemia (had postoperative HCT value of below 21%) (AOR: 0.092, 95%CI: 0.01, 0.956) (Table 2).

**Table 2:** Factors associated with maternal death secondary to uterine rupture (*n* = 242) in Debremarkos referral Hospital from 2011–2014

Variable	Maternal death		COR95%CI	AOR 95% CI	*P* value
Place of labor	Yes (%)	No (%)			
Home	14(5.8)	124(51.2)	5.76(1.28–25.92)*	6.92 (1.16–33.74)*	0.033
Health facility	2(0.8)	102(42.1)	1	1	
Hypovolemic shock					
Yes	10(4.1)	73(30.2)	3.49(1.22–9.98)*	3.48 (1.01–11.96)*	0.048
No Age	6(2.5)	153(63.2)	1	1	
≥34	3(2.9)	114(47.1)	0.24(0.03–2.02)	0.092 (0.01–0.956)*	0.046
22–33	5(0.4)	67(27.7)	2.9(0.99–8.45)	2.18 (0.06–7.81)	
≤21	8(3.3)	45(18.6)	1	1	

* = statistically significant at *P* – value < 0.05

## Discussion

The prevalence of uterine rupture was found to be 2.24% (1 in 41 deliveries) in the study area. This finding is higher as compared to the studies from east and west Nigeria (0.4%) [12] and (1.2%) [13], Ghana(1 in 124) [15], Turkey (1 in 287) [8], Saudi Arabia (1 in 10011) [9], Nordic region (5.6 per 10 000) [16]. In addition, it is higher that the WHO systematic review for the causes of maternal mortality secondary to uterine rupture (0.053%) [5], Pakistan (0.41%) [17], Bangladesh (0.67%) [18] and North West Nigerian (0.61%) [19]. This difference could be partly explained by the high prevalence of home delivery service (57%), socioeconomic and cultural differences across countries, availability and accessibility of skilled maternal care. Moreover, 85.5% of the mothers who suffered uterine rupture are from rural areas which limit the access to improved maternal care. It was also higher when compared with the study in Adigrat hospital, Ethiopia (0.91%) [14]. This might be due to the improved medical documentation practice, demographic variation and low antenatal care service utilization in the study area.

However, the prevalence in the current study was lower when compared with studies in Angola (4.9%) [20] and Yergalem general hospital, Ethiopia (5%) [21]. This could be due to the time variation, improvement of health service infrastructure and quality of health service provision.

In this study, 57% (138) of the mothers labored at home. This finding is consistent with the study in Adigrat hospital, Ethiopia [14]. The possible explanation could be due to lack of accessibility of adequate hospitals in rural parts of Ethiopia and lack of awareness of institutional delivery service utilization in most parts of the country [22].

Obstructed labor accounted for 89.3% (216) of uterine rupture in this study, while previous uterine scar accounted for only 3.7% (9) cases. This figure is much higher than studies from Nigeria, 47.3% (scar = 22.1%), Adigrat in the northern Ethiopia 79.6% (scar = 11.2%) and Pakistan 12.5% (scar = 12.5%) [12, 14, 17]. This difference might be due to the variation in socio- economic factors and environmental factors, lack of transport for immediate shifting of patients to the referral hospital illiteracy in the community, lack of antenatal care, lack of screening for high-risk pregnancy, and unsupervised labor conducted in poorly equipped centers. It might also be due to the inadequacy of the health facilities to recognize and give definitive management for Cephalopelvic disproportion (CPD) and obstructed labor.

The current study shows, the majority of uterine rupture, 208 (86%) have a complete uterine rupture and 34(14%) had incomplete rupture. Similar to this, finding, a study from Turkey by Turgut and colleagues revealed that complete type of rupture is more likely to occur among mothers admitted for the management of uterine rupture [23]. Hysterectomy was performed for 73.9% of patients (Total Abdominal Hysterectomy = 57% (138) and Subtotal hysterectomy (STAH) =16.9% (41)). For 27% of the cases, uterine repair (23.3% repair with BTL and for 3.7% repair without BTL) was performed. This finding is higher than studies from Turkey(TAH 0 34%) [23] and Nigeria, where hysterectomy was performed for 31.6% of cases (TAH =7.4% STAH = 24.2%) and repair without BTL was done for 30% of cases of uterine rupture [24]. The possible explanation could be the difference in health care provider’s skill and the difference in health institution set up [25]. Ruptured uterus is the most common indication in performing hysterectomy [26–28]. However, abnormal placentation was reported to be the most common indication for hysterectomy by a study from Turkey [8]. Although hysterectomy is considered as lifesaving procedure of obstetric emergency [26], it is invasive procedure [29]. Vaginal hysterectomy is rather minimally invasive and should be the first line of choice to consider before performing total hysterectomy [29]. If there is imminent uterine rupture around uterine fundal region, as Suhin and colleagues recommended, it might be essential to consider alternative second-line strategies (such as intrauterine balloon tamponade, uterine artery embolization and compression sutures) to minimize blood loss and preserve uterus [30]. However, identifying the site of uterine rupture without performing laparotomy is challenging in medical practice.

Comparable hysterectomy rate was reported from Pakistan where hysterectomy was done for 76.6% (TAH = 26.6%, STAH = 50%) [17]. This might be due to the similarity in obstetrics causes of uterine rupture. Comparable proportions of obstetric risk factors such as obstructed labor, prolonged labor and previous history of uterine scar were reported in the Pakistani study [17].

Blood availability and transfusion in catastrophic situations of uterine rupture is a lifesaving factor. As a result, this study shows 78% of the mothers who suffered from uterine rupture received blood transfusion which is comparable with the study conducted in Al-Batool Teaching Hospital, (83%) [7]. But in this study, more patients were transfused when compared with the study in Adigrat and in Yemen (57.1%), (59.3%) respectively [14, 31]. This could be probably due to the poor hemodynamic state in which patients arrived in health institutions.

The current study shows 50% of cases developed anemia postoperatively. Out of these, severe anemia accounts for 21.9% of cases, but this finding is lower than the finding from Nigeria (93.7%) [13]. The possible explanation might be due to the universal iron folate supplementation for all Ethiopian pregnant women by community health extension workers. Hence, this iron supplementation has a capacity to protect the occurrence of anemia after delivery whatever its complication level of labor would be [32]. In addition, low level of hemoglobin is associated with post-partum hemorrhage [33].

In 9.9% of the uterine rupture cases, there was associated bladder rupture which is comparable with a study from Yemen (8.6%) but lower than a study from Pakistan (21.1%) [10, 31]. Bladder rupture in association with uterine rupture reflects the presence of obstructed labor and late presentation of mothers to the hospital [34].

In this study, fourteen cases (5.8%) developed vesico-vaginal fistula and 0.4% of cases developed recto-vaginal fistula. This finding is slightly higher than the finding in Nigeria (4.2%) and Yirgalem (3%) [21, 24]. The possible explanation for this discrepancy could be due to the high occurrence of obstructed labor in this study and this is the main cause of rupture. But, the findings of uterine rupture in other studies may not be secondary to obstructed labor. The occurrence of bladder rupture and vesico-vaginal fistula suggest most of the rupture cases were attributed by prolonged and obstructed labor and late presentation of mothers to the Hospital [5, 7].

However, the proportion of vesico-vaginal fistula in this study is lower than the finding from Adigrat study (12.5%) [14]. The possible explanation is the occurrence of high prevalence of obstructed labor in the study area in association with uterine rupture and there was an association between bladder injury with obstructed labor [14].

From 2011 to 2014, 242 preventable cases of uterine rupture occurred in Debremarkos Hospital. Uterine rupture has been pointed out as a preventable tragedy of obstetric care in developing countries [35, 36]. It can, however, can be prevented by sustained ante-natal care institutional deliveries, early diagnosis, improved obstetric techniques [23, 34] and improving obstetric care to reduce the delay of women reaching obstetric clinics particularly for women with prolonged labor [35]. During the period between 2011 and 2014, 45 maternal deaths occurred in the hospital, 16(6.6%) of deaths include mothers managed for uterine rupture; this gave maternal mortality rate of 35.5%. This figure is comparable to the findings in Pakistan 7.8% and in Yirgalem 6% [17, 21]. However, it is higher than a study from Turkey (no maternal death was recorded) [23]. This is due to the difference in the infrastructure of hospitals and health care provider’s skill [25].

But, maternal mortality secondary to uterine rupture in this study was found to be lower than the study from Angola (13.6%), and Adigrat (11.1%) [14, 20]. The possible explanation for this might be an earlier presentation of mothers to the hospital set up, timely diagnosis of uterine rupture, adequate resuscitation of patients, availability of blood transfusion, absence of delay between diagnosis and definitive management and presence of experienced surgeon has effect decrement of maternal death after uterine rupture.

In this study, six (37.5%) patients died from hypovolemic shock, 4(25%) patients from severe anemia, 3 patients (18.7) from septic shock, 1 patient (6.3%) from acute renal failure and 1 patient (6.3%) from pulmonary edema. This figure is comparable to the study in Bangladesh where major causes of immediate death from uterine rupture were irreversible shock from severe hemorrhage and septicemia [18].

In addition, 87.5% maternal deaths were among a mother who labored at home and was found to be significantly associated with maternal death than laboring at a health facility. This study is similar to the study done in Angola where maternal death was higher among mothers who labored at home and referred from primary health unit [20]. This can be explained by mothers being in a more unstable condition are highly likely to die.

### Limitation of the study

Twelve charts of women managed for uterine rupture could not be retrieved or were incomplete. Hence, these charts were not included in the study.

## Conclusion

The magnitude of uterine rupture was high in the study area. Initiation of labor at home, the occurrence of hypo-volumia shock and occurrence of post-operative severe anemia were factors significantly associated with maternal death secondary to uterine rupture. Early diagnosis, well equipped Intensive Care Unit (ICU), good blood bank service, Neonatal Intensive Care Unit (NICU), can help reduce maternal and perinatal mortality secondary to uterine rupture. In addition, initiation of labor in a health institution, early treatment of hypo-volumia and prevention of anemia is essential to decrease maternal death secondary to uterine rupture.

## Abbreviations

ANC: Ante natal care
AOR: Adjusted odds ratio
BTL: Bilateral tuba ligation
COR: Crude odds ratio
CS: Cesarean section
FMOH: Federal ministry of health
HCT: Hematocrit
ICU: Intensive care unit
MMR: Maternal mortality ratio
NICU: Neonatal intensive care unit
SAH: Subtotal abdominal hysterectomy
SBAs: Skill birth attendants
SPSS: Statistical package for social science
TAH: Total abdominal hysterectomy
WHO: World Health Organization
